# Severe Fungal Asthma: A Role for Biologics and Inhaled Antifungals

**DOI:** 10.3390/jof9010085

**Published:** 2023-01-06

**Authors:** Richard B. Moss

**Affiliations:** Center of Excellence in Pulmonary Biology, Division of Pulmonary, Asthma and Sleep Medicine, Department of Pediatrics, Stanford University School of Medicine, 770 Welch Road, Suite 350, Palo Alto, CA 94304, USA; rmoss@stanford.edu

**Keywords:** allergic bronchopulmonary aspergillosis (ABPA), *Aspergillus fumigatus* (*Af*), asthma, biologicals, amphotericin, azoles, monoclonal antibodies (Mab), severe asthma with fungal sensitization (SAFS), type 2 immune deviation disease (T2)

## Abstract

Allergic asthma has traditionally been treated with inhaled and systemic glucocorticosteroids. A continuum of allergic fungal airways disease associated with *Aspergillus fumigatus* colonization and/or atopic immune responses that encompasses fungal asthma, severe asthma with fungal sensitization and allergic bronchopulmonary aspergillosis is now recognized along a phenotypic severity spectrum of T2-high immune deviation lung disease. Oral triazoles have shown clinical, anti-inflammatory and microbiologic efficacy in this setting; in the future inhaled antifungals may improve the therapeutic index. Humanized monoclonal antibody biologic agents targeting T2-high disease also show efficacy and promise of improved control in difficult cases. Developments in these areas are highlighted in this overview.

## 1. Introduction

It is useful to situate the patient with allergic fungal airways disease within the larger context of fungal lung disease, with a particular focus on *Aspergillus fumigatus* (*Af*) as a single species; although others may be involved, this is the main pathogen to be concerned about in the vast majority of cases. Inhalation of *Af* spores is ubiquitous and universal. In a normal healthy human host with no underlying problems arising from the lungs or the immune system, these spores are disposed of by innate host defenses without any sequelae. However, if there is an underlying immunodeficiency or functional/structural lung disease then a variety of lung conditions can occur [1]. In the case of asthma, this disease can be considered one of a group of muco-obstructive lung diseases that provide a permissive endobronchial environment for the potential germination and persistent proliferation of thermotolerant fungi in association with mucus plugs, mucoid impaction and, in severe cases, concomitant bronchiectasis.

## 2. The Allergic Fungal Airways Disease Concept

**Figure 1:**
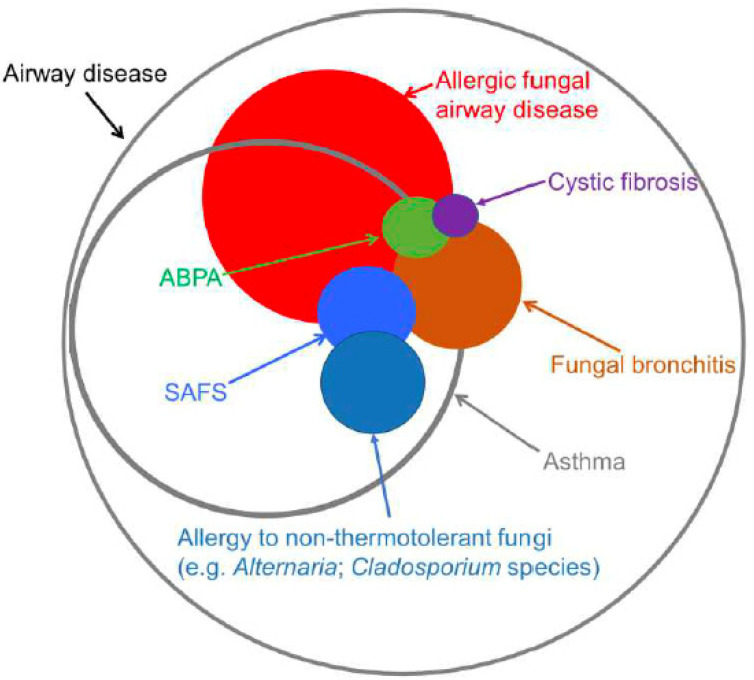
Concept of an overlapping spectrum of allergic fungal airway diseases. Adapted from reference [7]. ‘Journal of Asthma and Allergy 2021:14 557–573’, originally published by and used with permission from Dove Medical Press Ltd.

While there are several recognizable phenotypes of allergic fungal airways disease, there are several common pathogenetic features to this spectrum of related conditions including allergic bronchopulmonary aspergillosis (ABPA) and severe asthma with fungal sensitization (SAFS) (Figure 1) [2,3,4,5,6,7]. The phenotypes emerge from immune responses to fungi capable, like *Af*, of adapting to the intraluminal airways environment due to several features including thermotolerance (ability to grow at human body temperature) and elaboration of several structural or metabolic products such as potent proteases that affect the respiratory epithelium, either damaging it, activating it or both. These features, along with adaptation to nutritional, oxidative and hypoxic stressors existing in the airways, allow fungal survival and orchestrate an immune-deviated T_H_2 type response in the lungs. Responses are likely to occur initially through innate immunity by activation of innate lymphoid type 2 cells (ILC2) and later in the adaptive arm of immunity by activation of certain dendritic cells to produce T_H_2-skewing chemokines such as CCL17 that direct CD4+ T cells to differentiate to a T_H_2 phenotype. ILC2 and CD4+ T_H_2 cells both orchestrate a suite of responses, largely dependent upon canonical T_H_2 cytokines IL-4, IL-5 and IL-13 production, prominently featuring eosinophilic infiltration and activation but also B cell isotype switch to IgE production, and mast cell and basophil activation, all of which contribute to clinical phenotypes of varying severity allergic fungal asthmatic airways disease [8,9].

**Figure 2:**
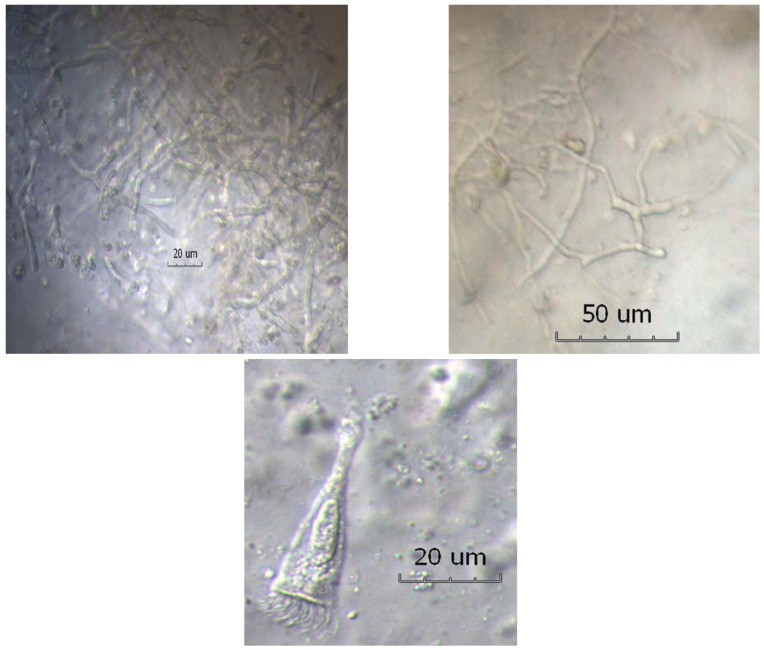
Persistent non-invasive endobronchial growth of thermotolerant fungi, particularly *Aspergillus fumigatus* (*Af*), occurs in mucus plugs seen in muco-obstructive diseases (here, in an expectorated sputum sample from a patient with cystic fibrosis and allergic bronchopulmonary aspergillosis). Upper panels show typical branching *Af* hyphae. The lower panel shows T2 inflammatory response with a desquamated ciliated epithelial cell surrounded by eosinophilic granulocytes, some of which appear to have degranulated. Adapted from reference [12]. Reproduced with permission of the © ERS 2023.

It is worth emphasizing the capabilities of *Af*, one of ~200 *Aspergillus* species and of literally millions of other species in the fungal Kingdom, that make it such a dominant fungal pathogen in muco-obstructive lung diseases. *Af* is one of only a few thermotolerant fungal species capable of good growth (in fact, optimal) at body temperature. Conidia of *Af* are hydrophobic and thus both highly dispersible and respirable to the lower airways with a mass median diameter of 2–4 µM. They are coated with an immunologically inert rodlet layer, and contain melanin that protects against oxidants as well as sialic acid moieties and cell wall components that contribute to strong adhesion to respiratory epithelium. *Af* has a large genome of ~10,000 genes (half the size of the human genome) that encodes proteins facilitating immune evasion such as biofilm formation; coordinated stress responses to limited oxygen, iron, glucose and nitrogen sources; adhesion (e.g., by galactosaminogalactan) and cytotoxicity (gliotoxin) [10,11]. In muco-obstructive lung diseases such as severe asthma, but also cystic fibrosis and non-CF bronchiectasis, ciliary dyskinesia and severe chronic obstructive pulmonary disease (COPD), *Af* is able to grow within mucus plugs into mycelial mats which are very effective in producing strong T_H_2 responses, resulting in endobronchial and mucosal eosinophilic inflammation discernable in expectorated sputum as well as peripheral blood eosinophilia (Figure 2) [12,13].

It has been recognized that T_H_2 airway responses exist along a range of recognized clinical phenotypes ranging from nonfungal allergic asthma to asthma associated with fungal sensitization (which may be caused by exposure to non-thermotolerant non-colonizing fungi such as *Alternaria* species), SAFS, ABPA without bronchiectasis (i.e., serologic ABPA) and ABPA with bronchiectasis. Of the approximately 10% of clinical asthma categorized as severe by American Thoracic Society/European Respiratory Society or Global Initiative for Asthma definitions [14,15], epidemiologic surveys have reported that 20–50% are associated with fungal sensitization phenotypes along this spectrum [16,17,18]. Clinically, there is now extensive international evidence that *Af* sensitization in particular is associated with a more severe and recalcitrant asthma phenotype, including greater airflow limitation, bronchiectasis, mucus plugging, clinical morbidity, diagnosed SAFS and ABPA, in both children and adults [19,20,21,22,23,24,25,26,27,28,29].

**Figure 3:**
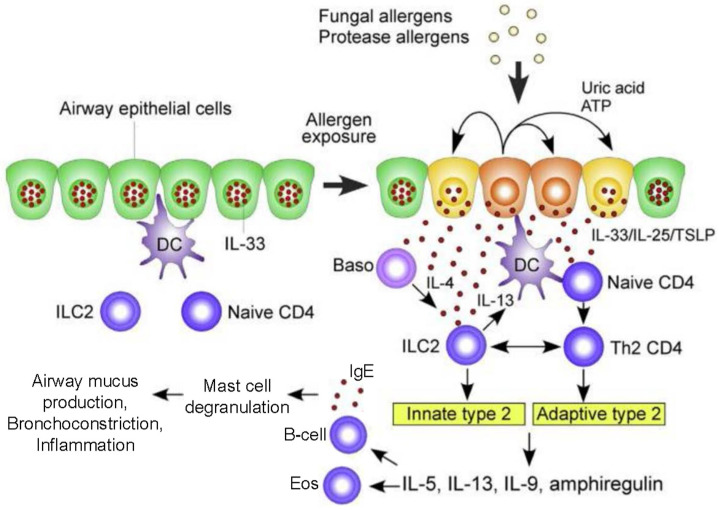
Immunopathogenesis of allergic bronchopulmonary aspergillosis. In the airways of susceptible people with muco-obstructive lung disease, growth of thermotolerant *A. fumigatus* yields pathogen-associated molecular patterns that are recognized by epithelial pattern recognition receptors of the innate arm of immunity to activate epithelium to produce alarmins and subsequently type 2 innate lymphoid cells to produce a T2-deviated cytokine response, while fungal allergens engage mucosal dendritic cells to attract and activate Th2 CD4+ cells to produce a similar T2-dominant adaptive immune response. Both arms of the T2-high immune response orchestrate IgE production, mast cell and basophil activation, and a predominantly eosinophilic luminal and mucosal inflammatory infiltrate. Source: Reference [33].

ABPA is the most common severe fungal asthma phenotype bringing patients to subspecialty care, with approximately five million patients estimated worldwide [30,31,32]. In ABPA, fungal products, including a variety of proteases, chitin, gliotoxin and other substances, act upon the respiratory epithelium to produce alarmins including IL-33, TSLP and IL-25, which activate innate and adaptive T_H_2-skewed responses prominently featuring eosinophilic inflammation as well as polyclonal IgE production and mast cell and basophil activation (Figure 3) [33]. Recently the role of galectin-10, aggregates of which form Charcot–Leyden crystals (identified over 150 years ago in asthma and prominently found in ABPA endobronchial mucus plugs), has been recognized as a possible key component of pathology and potential novel therapeutic target [34,35]. 

ABPA was recognized as a distinct disease in the early 1950s [36]; for nearly 70 years the mainstay of therapy has relied upon the potent anti-inflammatory properties of systemic glucocorticosteroids [37]. However, while oral steroids are effective in inducing remission, side effects have proven highly problematic, as prolonged, and for many patients repeated, treatment courses are required for disease control. Additionally, the possibility of steroid therapy increasing vulnerability to invasive aspergillosis, as occurs in several lung diseases such as severe COPD and COVID-19 in otherwise immunocompetent individuals, needs to be considered. High-dose “pulse” (monthly) intravenous methylprednisolone infusions have thus achieved some popularity as a possibly less toxic steroid treatment alternative [38], but inconvenience, access, cost and continued side effect issues have limited their usefulness.

## 3. Oral Azole Therapy for Allergic Fungal Airways Disease

In the 1990s the availability of *Af*-active oral triazole agents, beginning with itraconazole and now including voriconazole, posaconazole and isavuconazole, added a sorely needed adjunct or alternative to steroid therapy for ABPA [39,40]. Controlled trials have established the effectiveness and acceptable safety profile of azole therapy for ABPA, particularly itraconazole as it is the oldest and most widely used therapeutic azole [41,42,43,44,45,46]. The main benefits seem to reside in reduction of exacerbation rates and steroid exposure. However, here again there is a problem with specific azole toxicities largely due to the requirement for prolonged and repeated treatment courses of at least several months duration. Achieving therapeutic drug levels can be difficult due to reduced absorptive bioavailability (mainly a problem with itraconazole), pharmacogenomic variation in the recipient population affecting metabolism and effects of concomitant drug use. Other pharmacodynamic complications involve drug–drug interactions arising from hepatic metabolism of azoles affecting other therapeutic drug levels or being affected by them [40,47]. Important examples include azoles reducing clearance of some systemic and inhaled glucocorticoids that may be needed to treat asthma, and of the CFTR modulator ivacaftor needed to treat cystic fibrosis. Additionally, the problem of increasing prevalence of azole resistance, associated with therapeutic failure, is now recognized internationally [48]. Importantly, there remains a dearth of high-quality controlled clinical trials and guidance on the relative merits and risks of front-line azole versus oral steroid therapy, whether these drugs should be employed alone or together and in which various clinical stages of ABPA [49,50,51,52]. Finally, controlled trials of oral azoles in SAFS have yielded conflicting results, possibly due to different agents (itraconazole or voriconazole) and treatment duration (8 months or 3 months) [53,54].

## 4. Inhaled Antifungals for Allergic Fungal Airways Disease

Problems with systemic azole therapy have largely driven interest in other antifungal alternatives. The polyene antifungal agent amphotericin B is highly active against *Af* but also as an intravenous agent is prohibitively nephrotoxic for required prolonged clinical use in allergic (non-invasive) muco-obstructive lung disease. This has led to exploration of off-label inhalational use of several intravenous formulations of amphotericin going back half a century, tried in various doses, regimes and aerosol delivery systems [55,56]. However, most of this experience arose in the context of immunosuppressed patients (e.g., lung transplant recipients, neutropenia, hematologic malignancy) at risk of or suffering from invasive aspergillosis or other mycoses. Recent studies of inhaled amphotericin in asthmatic subjects with ABPA have found decreased exacerbations over time, accompanied by biomarker evidence of subsiding T_H_2 inflammatory responses. These results suggest this approach has some merit but probably insufficient impact to be widely adopted, particularly when side effects like bronchospasm associated with parenteral formulation are considered [57,58,59].

The potential of antifungal therapy delivered directly to the airway exemplified by efforts to repurpose intravenous formulations has led to nascent development of newly formulated antifungal agents, sometimes accompanied by newly designed aerosol delivery devices. Itraconazole, for example, has been reformulated as a respirable dry powder and bundled with a dedicated inhaler for efficient delivery of drug preferentially to the conducting airways (PUR1900, Pulmatrix, Lexington MA, USA). Early phase clinical results documented sputum levels ~70 times higher and plasma levels 100–400 times lower compared with conventional oral itraconazole dosing. Sputum levels exceeded *A. fumigatus* MIC_90_ (500 ng/mL) in most cases for up to 24 h [60]. A 16-week safety and preliminary efficacy phase 2 trial in asthmatics with ABPA is expected to begin enrollment in 2023 [61]. A novel inhaled azole agent (PC945, Pulmocide, London, UK) has been developed for nebulization delivery [62], with a first indication for clinical licensing in salvage therapy of invasive pulmonary aspergillosis. It is also well tolerated in asthmatics, raising the potential of application to allergic fungal airway disease [63]. Further efforts at development of dedicated inhaled antifungals, including other azoles and amphotericin, are underway but not yet in clinical stage development.

## 5. Biological Agents for Allergic Fungal Airways Disease

Treatment of severe asthma has been revolutionized by the recognition that a majority of cases are associated with T_H_2 or type 2 immune deviation (T2) and a subsequent largely eosinophilic inflammation, as described earlier. Advances particularly in humanized monoclonal antibody (Mab) technology over the past two decades have resulted in a current roster of six approved Mabs raised against several T2 targets: omalizumab for immunoglobulin E; mepolizumab, reslizumab and benralizumab for the cytokine interleukin-5 or its receptor; dupilumab for the interleukin-4 receptor α, critical for signal transduction producing both IL-4 and IL-13; and tezepelumab for thymic stromal lymphopoietin (TSLP), an epithelial alarmin. Clinical data have slowly accumulated for use of four of these agents (omalizumab, mepolizumab, benralizumab, dupilumab) specifically in the setting of ABPA (summarized in Table 1). In a comprehensive review encompassing qualitative analysis of 49 studies (*n* = 267) and quantitative meta-analysis of 14 case series (*n* = 167), omalizumab for ABPA significantly (a) reduced annualized exacerbation rate versus pre-treatment, (b) reduced oral steroid use and dose, (c) increased wean off steroids, (d) improved lung function and (e) improved asthma control [64]. In the aggregate, most patients receiving a T2-targeting Mab for ABPA showed reduction in exacerbation rate and a steroid-sparing effect along with reduction in total IgE and eosinophila, with less consistent effects on lung function and patient-reported outcome instruments [64,65,66,67]. In several cases that were reported with serial chest computed tomographic imaging, pulmonary infiltrates—presumed eosinophilic mucoid impaction and mucus plugs associated with *Aspergillus* sensitization and more severe asthma phenotype [29]—cleared soon after initiation of Mab treatment [68,69,70,71,72,73].

Interpreting biomarker data for evidence of Mab clinical efficacy in asthma can be applied to ABPA and SAFS. For example, omalizumab’s clinical effectiveness in reducing asthma exacerbation rate is reflected in the biomarkers forced exhaled nitric oxide (F_E_NO) and blood eosinophilia, where higher values for each are seen in those patients who have greater reductions in exacerbation rate on treatment [74]; these biomarkers behave similarly in ABPA, consistent with the concept that ABPA (and SAFS) reflect more severe phenotypes of the allergic fungal asthma disease spectrum [75,76].

Several conclusions from recent reviews can be summarized as follows: first, most data come from descriptive studies (cases and series) rather than controlled trials and the bulk of the data exists for the Mab in longest use for allergic forms of asthma, omalizumab. Second, despite the inherent danger of selection and publication bias there is strong uncontrolled evidence for positive effects on several clinical outcome measures and associated biomarkers. Third, enough published cases exist demonstrating positive responses after switching from one biologic to another in the face of an initial Mab’s lack of clinical efficacy that switching after a trial of one Mab to another is a defensible tactic, although the data are too sparse to discern any comparative Mab utility overall [73,77,78]. Fourth, the lack of high-quality clinical evidence (except for a positive randomized double-blind placebo-controlled crossover trial of omalizumab in subjects with asthma and ABPA [75]) strongly argues for the conduct of appropriate well-designed randomized placebo-controlled trials to validate and help generalize these conclusions.

**Figure 4:**
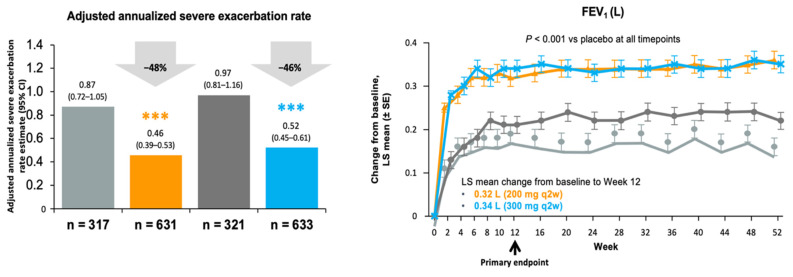

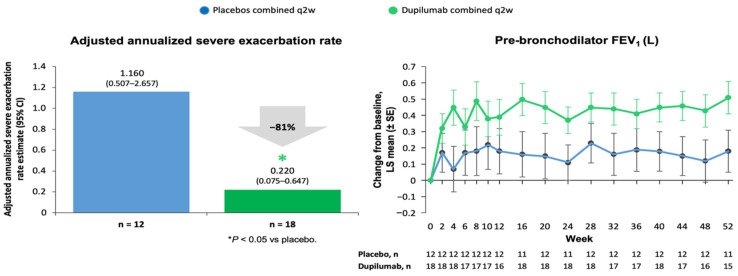
Clinical outcomes of annualized severe asthma exacerbation rate (*left panels*) and change in lung function (*right panels*) in a randomized, double-blind, placebo-controlled one-year pivotal trial of dupilumab in adult patients with moderate-severe asthma. *Top panel*: Results of overall study. Dupilumab reduced asthma exacerbations by 46–48% and improved lung function (*** *p* < 0.001) in both dosage arms of active treatment compared to placebo. *Bottom panel*: Post hoc analysis of study subjects with serologic ABPA (IgE > 1000 IU/mL, Af-specific IgE > 0.35 IU/mL, eosinophil count > 500) showing pooled results for actively treated subject arms compared to placebo. Dupilumab treatment reduced exacerbations and improved lung function to an even greater degree than seen in the overall study results. Adapted from references [80,82].

Recently, data have emerged from clinical trials and real-world effectiveness studies of dupilumab that supports its potential in treating ABPA based on a subset of severe asthma patients selected for pivotal trial inclusion on phenotypic characteristics [79,80]. As seen in analyses of omalizumab effectiveness in asthma, dupilumab’s clinical benefit is greater in subjects with more pronounced T2 biomarkers (F_E_NO, eosinophils) compared with asthmatics lacking a T2 biomarker signature or more modest elevations in these biomarkers [80,81]. A post hoc analysis of data collected in one of the pivotal phase 3 trials of dupilumab (Liberty Asthma Quest) [80] compared subjects meeting serologic criteria for ABPA (total IgE > 1000 IU/mL, *Aspergillus*-specific IgE > 0.35 IU/mL, eosinophils > 500) receiving dupilumab (*n* = 18) to those receiving a placebo (*n* = 12) [82]. Baseline characteristics were well matched. Dupilumab significantly reduced severe asthma exacerbations and improved lung function in the seropositive ABPA group on the drug compared to the placebo, and also to a greater degree than in the large sample of non-ABPA asthmatics in the trial (Figure 4), while also significantly reducing total and *Aspergillus*-specific IgE levels, F_E_NO and CCL17/TARC levels [82]. However, the highest quality evidence rests upon randomized placebo-controlled trials designed to specifically address the question of efficacy and safety in a well-defined target population sample, leading to a licensing label expansion. Fortunately, such a trial has been initiated for the use of dupilumab in subjects with asthma and ABPA (https://clinicaltrials.gov/ct2/show/NCT04442269, URL accessed on 4 December 2022; Regeneron-Sanofi).

Are biologics useful in SAFS as well as ABPA? If the T2 spectrum concept holds for allergic fungal airway disease this should be the case. Recent studies suggest that T2-targeting biologics do indeed have positive effects on SAFS. In one, a 24-month study including asthmatics with SAFS (*n* = 62) or ABPA (*n* = 11), omalizumab treatment resulted in similarly favorable clinical endpoint outcomes (exacerbation frequency, oral steroid use, Asthma Control Questionnaire-5 scores) in SAFS and ABPA subjects as in asthmatics without fungal sensitization [83]. In another, improved asthma clinical outcomes (asthma control, exacerbations, steroid use) in SAFS patients (*n* = 193) treated with mepolizumab (*n* = 63) or benralizumab (*n* = 130) were also positive and similar to outcomes in non-fungal sensitized severe asthmatics [84].

## 6. Conclusions

Asthma phenotypes associated with fungal sensitization including SAFS and ABPA are likely to represent T2-high immune response endotypes where the persistent presence of thermotolerant fungi (including *Aspergillus* but also *Penicillium* and *Mucor* species [85,86]) growing in airway mucus plugs perpetuate and worsen airway inflammation with resulting clinical morbidities. A T2-high phenotype does not exclude additional immunopathologic mechanisms; for example, the T_H_17 immune response to *C. albicans* has been shown to give rise to a subset of cross-reactive cells, recognizing *A. fumigatus* epitopes that expand during ABPA flares [87,88]. Fungal allergic sensitization is an operative factor in 20–50% of severe asthma cases, representing in turn 5–10% of all worldwide asthma. Basic and clinical research indicates that this huge illness burden can be addressed by efforts to reduce fungal airway burden with antifungal therapy, which is evolving towards direct topical airway application by development of dedicated inhalational antifungals. It can also be improved by careful use of biologic Mabs targeting T2 immune response pathways including IgE, IL-5, IL-4 and IL-13 in difficult-to-control allergic fungal airway disease. Further studies may extend these targets upstream in the pathogenic pathway to include epithelial alarmins such as TSLP [89]. Properly designed clinical trials are necessary to validate the promise of these new approaches to asthma treatment in people with allergic fungal airway disease.

## Figures and Tables

**Table 1:** Aggregate summarized clinical outcome results of 59 studies with 228 adult patients treated with biologics for allergic bronchopulmonary aspergillosis. Source: Reference [67].

Biologic Agent	Studies Reviewed	Patients Included	Reduction in Total IgE	Reduction in Eosinophil Count	Reduction in Exacerbation Rate	Reduction in Steroid Use
Omalizumab	36	133	45%	70%	90%	66%
Mepolizumab	13	62	67%	95%	85%	98%
Benralizumab	6	7	40%	99%	90%	95%
Dupilumab	4	26	65%	48% *	85%	90%

* following transient initial increase.

## Data Availability

Data extracted from cited literature.
