# Correction: Moss, R.B. Severe Fungal Asthma: A Role for Biologics and Inhaled Antifungals. *J. Fungi* 2023, *9*, 85

**DOI:** 10.3390/jof9040450

**Published:** 2023-04-06

**Authors:** Richard B. Moss

**Affiliations:** Center of Excellence in Pulmonary Biology, Division of Pulmonary, Asthma and Sleep Medicine, Department of Pediatrics, Stanford University School of Medicine, 770 Welch Road, Suite 350, Palo Alto, CA 94304, USA; rmoss@stanford.edu

In the original publication [1], the author would like to add copyright permission in the legend of Figure 1. It should be updated as below:

“**Figure 1.** Concept of an overlapping spectrum of allergic fungal airway diseases. Adapted from reference [7]. ‘*Journal of Asthma and Allergy* 2021:14 557–573’, originally published by and used with permission from Dove Medical Press Ltd.”

The original publication has also been updated.
